# Exploring Conformational Transitions in Biased and Balanced Ligand Binding of GLP-1R

**DOI:** 10.3390/molecules30153216

**Published:** 2025-07-31

**Authors:** Marc Xu, Horst Vogel, Shuguang Yuan

**Affiliations:** 1 Research Center for Computer-Aided Drug Discovery, Shenzhen Institutes of Advanced Technology, Chinese Academy of Sciences, Shenzhen 518055, China; 2 University of Chinese Academy of Sciences, Beijing 100049, China; 3 Faculty of Pharmaceutical Sciences, Shenzhen University of Advanced Technology, Shenzhen 518055, China; 4 Institut des Sciences et Ingénierie Chimiques (ISIC), Ecole Polytechnique Fédérale de Lausanne (EPFL), 1015 Lausanne, Switzerland; 5 AlphaMol Science Ltd., Shenzhen 518055, China

**Keywords:** GLP-1R signaling, conformational dynamics, structural analyses, molecular dynamics simulation

## Abstract

The glucagon-like peptide-1 receptor (GLP-1R), which belongs to the class B1 G protein-coupled receptor (GPCR) family, is an important target for treatment of metabolic disorders, including type 2 diabetes and obesity. The growing interest in GLP-1R-based therapies is driven by the development of various functional agonists as well as the huge commercial market. Thus, understanding the structural details of ligand-induced signaling are important for developing improved GLP-1R drugs. Here, we investigated the conformational dynamics of the receptor in complex with a selection of prototypical functional agonists, including CHU-128 (small molecule-biased), danuglipron (small molecule balanced), and Peptide 19 (peptide balanced), which exhibit unique, distinct binding modes and induced helix packing. Furthermore, our all-atom molecular dynamics (MD) simulations revealed atomic feature how different those ligands led to signaling pathway preference. Our findings offer valuable insights into the mechanistic principle of GLP-1R activation, which are helpful for the rational design of next-generation GLP-1R drug molecules.

## 1. Introduction

G protein-coupled receptors (GPCRs) are important mediators of extracellular signals across the cell’s plasma membrane, conveying external stimuli to intracellular effectors that regulate various cellular processes. The activation process of GPCRs is driven by an internal network of interacting residues, which coordinates conformational transitions between the functional states of the receptor. Structural elucidation of receptor–ligand complexes has provided valuable insights into important interaction networks that trigger specific signaling pathways [1]. Recent advances in the determination of GPCR structures have significantly enhanced our understanding of receptor functions and fostered the structure-based drug discovery of GPCR drugs. Yet, experimentally determined GPCR structures often fail to capture the full inherent flexibility of GPCRs, as they typically represent a very limited number of static snapshots of the receptor’s large dynamic conformational landscape in the activation process [2]. To better understand the receptors’ functional transitions and conformational dynamics, the static structures of the receptor in specific activation states serve as a constitutive basis for in silico simulation of the entire GPCR activation process [2,3,4].

In recent years, glucagon-like peptide 1 (GLP-1) receptor have gained considerable attention as a target for the clinical treatment of metabolic disorders, particularly of type 2 diabetes (T2D) and obesity. GLP-1R belongs the secretin-like family of class B GPCRs, which is predominantly expressed in pancreatic β-cells. It controls both insulin and glucagon secretion in glucose-dependent manner [5]. Activation of GLP-1R induces insulin secretion, inhibits glucagon secretion, and slows gastric emptying, thus contributing to the central regulation of blood glucose levels. Dysfunctional GLP-1R activation disrupts metabolic homeostasis, contributing to the pathogenesis of metabolic diseases [6]. However, the administration of GLP-1R agonists (GLP-1RAs) in T2D patients has been shown to improve glycemic control [7,8,9,10].

Over the past few decades, numerous GLP-1RAs, such as semaglutide and liraglutide, have been developed, featuring intrinsic modification of the native GLP-1 peptide to imitate the pharmacological profile of GLP-1, while providing resistance to degradation by the enzyme dipeptidyl peptidase-4 [11,12,13]. Interestingly, these molecules have demonstrated extended therapeutic benefits, including potential applications in cardiovascular diseases and neurodegenerative diseases, as well as in non-alcoholic fatty liver disease [6,14,15,16]. Despite their efficacy, some GLP-1RAs exhibit reduced therapeutic effects due to undesired receptor trafficking, attenuating the glucoregulatory responses by desensitization and downregulation caused by β-arrestin recruitment [17,18]. To overcome this limitation, the development of G_s_-biased agonists has been pursued; they enhance insulin release in cell-based experiments by selectively amplifying cAMP signaling [19]. Further evidence has been delivered showing that the administration of G_s_-biased agonists is more effective than cAMP and β-arrestin 2 signaling balanced agonists in regulation glucose homeostasis and bodyweight control in mice [20,21]. However, additional clinical studies are essential to fully evaluate the long-term implications of selective signaling modulation [22].

Furthermore, peptide GLP-1RAs are typically administrated via injection, which leads to challenge of patient compliance, gastrointestinal side effects, and production costs [23,24,25,26]. Consequently, the development of oral small-molecule agonists has emerged as an alternative approach, offering advantages such as improved patient compliance and bioavailability through oral administration [27,28,29]. These developments have prompted further investigation into the elucidation of experimental structure of activate state GLP-1R-G_s_ complexes with or without orthosteric agonists [30,31,32,33], providing crucial insights into the activation mechanisms induced by different ligands.

Despite significant progress in understanding the activation mechanisms of GLP-1R, structural insights into ligand-induced modulation by balanced and biased agonists remain limited. In this study, we aim to provide molecular insights into the different functional agonists binding to GLP-1R. Specifically, we investigate three ligands Peptide 19, danuglipron, and CHU-128 using all-atom model molecular dynamics (MD) simulations (Table 1). They show distinct functional features: (1) Peptide 19 is an analog of gastric inhibitory polypeptide (GIP) exhibiting high potency for cAMP production at both GLP-1R and GIPR, but with notable partial agonism toward β-arrestin 2 recruitment [34]. (2) Danuglipron is a small molecule identified from high-throughput screening and shows potency comparable to GLP-1 the canonical GLP-1R peptide in in vitro studies, inducing both cAMP and β-arrestin signaling [28]. In a phase 2 trial, the administration of danuglipron improved metabolic function, although gastrointestinal side effects were observed [35]. (3) CHU-128 is a small molecule that exhibits strong bias toward the cAMP pathway and distinct binding mode profile compared to danuglipron [30]. All mentioned ligands induce unique conformational changes in the receptor, resulting in distinct packing arrangement of the 7TM helical bundle that influences the downstream protein sensitivity to the intracellular binding pocket [30,31].

Using all-atom MD simulations of the ligand–GLP-1R–G_s_ complex, we explore how orthosteric-bound binders induce the transduction of signals across the receptor to activate specific or impartial intracellular signaling pathways. Furthermore, we perform complementary simulations with peptide-free GLP-1R to gain deeper insights into the receptor conformational dynamics, particularly in the extracellular domain, which is critical for agonist binding and activation. Our simulations reveal that the ligand binding mode is directly correlated to the signaling pathway preference. Importantly, the limitations of small molecules in simultaneously managing the extracellular domain (ECD), transmembrane region, and core vestibule reflect their distinct advantages in conducting GLP-1R activation.

## 2. Results and Discussion

### 2.1. Distinct Binding Mode Led to Unique Signaling

To understand how the conformation, orientation, and binding mode of ligands with distinct pharmacological profiles influence the receptor’s signaling pathway, we performed all-atom MD simulations for high-resolution structures of GLP-1R bound to different orthosteric ligands: (i) CHU-128 is a biased nonpeptide agonist that induces a receptor conformation that favors G protein activation over β-arrestin recruitment signaling [30]; (ii) danuglipron and (iii) Peptide 19 are a balanced nonpeptide and peptide agonist, respectively, which induce both cAMP production and β-arrestin recruitment pathways [30,31]. All these structures were simulated in complex with the G_s_ protein α-subunit (G_α_) only (Table 2). To visualize the potential mechanism that promotes the receptor to transit from one set of conformational states populations to another one, we performed an additional ligand-free MD simulation using a GLP-1R–Peptide 19 complex by removing the ligand and G_α_. Three independent replicas’ simulations of 500 ns duration were performed for each above-mentioned system.

**Figure 1:**
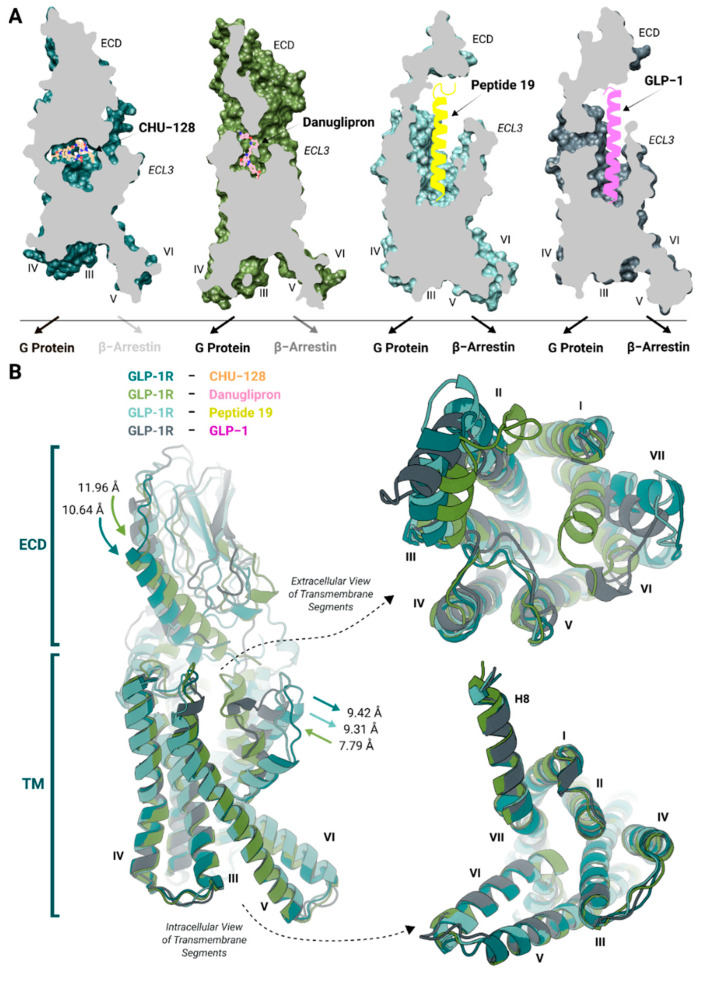
Structural comparison of GLP-1R bound to different agonists. (**A**) Cross-section view of experimental GLP-1R–ligand–G_s_ complex structures depicting CHU-128 (blue: receptor; orange: ligand), danuglipron (green: receptor; pink: ligand), Peptide 19 (cyan: receptor; yellow: ligand), and GLP-1 (black: receptor; purple: ligand) in their respective orthosteric binding pocket of GLP-1R. The transparency degree of downstream proteins reflects the relative potency and downstream signaling capacity of each ligand. (**B**) Superposition of GLP-1R structures comprising different bound ligands. The interface of the TM6-ECL3-TM7 region experiences significant displacement during receptor activation. Superposition of ligand-bound receptor structures reveals extracellular and intracellular conformational modulation, resulting in the formation of a similar intracellular cavity conformation among different types of ligands. The computed movements of agonist-bound structures are measured by comparison of the helix structure of the ECD and ECL3 regions to the GLP-1-bound structure. The Roman numbers from I to VII indicate TM segments of the receptor.

(i) The location of each ligand bound to the receptor. Static structural analyses of ligand–receptor complexes indicate that danuglipron inserts deeply in the receptor’s binding cavity closing the binding pocket, whereas CHU-128 adopts a planar binding pose above the helical transmembrane bundle, close to the extracellular domain (ECD), extracellular loop 1 (ECL1), and ECL2 liberating regions around TM6-TM7 [30] (Figure 1A). The position and binding modes of small-molecule agonists involve different structural modulations associated with distinct pharmacology profiles. Peptide agonists are integrally buried with a longitudinal orientation in the receptor’s binding vestibule, forming a helical structure within the transmembrane segments [36] (Figure 1). Either balanced or biased small-molecule agonists form a limited number of interactions with the core vestibule, suggesting that the extracellular interface plays a more critical role in signaling preferences than the buried region of the binding pocket. The contact with different transmembrane segments drives biased and balanced agonists to induce similar G protein-bound conformations at the intracellular binding site, thereby regulating the binding affinity of downstream proteins. Effectively balanced agonists confer the receptor a greater degree of conformational flexibility, shifting the intracellular binding site from a large, open to more restrained state [37].

(ii) Movement of TM region in small molecule-bound systems. The binding position of danuglipron toward the upper region of TM6-ECL3-TM7 prompts this region to shift inward within the binding pocket, promoting an overall contraction of the receptor structure compared to the GLP-1-bound structure [30] (Figure 1B). This contraction is crucial for mimicking the GLP-1-induced active receptor conformation. In contrast, no inward movement of these regions was observed for the CHU-128-bound structure, which instead shows a more extended conformation compared to peptide-bound structures. These distinct structural changes contribute to the pharmacological activity of the receptor, which is driven by modulating the affinity for downstream signaling proteins [38].

(iii) Structural differences in extracellular regions. There are noticeable differences in the structure of the receptor’s extracellular regions upon binding to different ligands. Peptide 19 induced a 9.31 Å outward movement of TM6-ECL3-TM7 relative to the GLP-1-bound structure (Figure 1B). This shift may stabilize the binding of the ligand in the core vestibule [39]. While GLP-1 engages stable interactions with the core vestibule but dissociates rapidly, Peptide 19 forms transient interactions in this domain with sustained interactions with the ECD and extracellular region of transmembrane helices, contributing to more durable G protein activation [31]. Unlike a typical class A binding mechanism, here the ECD plays a fundamental role in stabilizing the bound ligand [40]. Danuglipron induces a 7.79 Å inward movement of the TM6-ECL3-TM7 region, and the helix structure of the ECD shifts by 11.96 Å into the binding pocket. This movement reinforces the helical bundle’s compaction. Similar to danuglipron, CHU-128 induces a 10.64 Å inward shift in the helix structure of the ECD. Actually, the conformational modulation of the ECD is highly dependent on the particular ligand structure. Interestingly, CHU-129 induces a TM6-ECL3-TM7 shift comparable to that induced by Peptide 19. This region is associated with signaling pathways, highlighting the nuanced structural and functional differences between these ligands. In the structure of receptor bound to small-molecule agonists like danuglipron, the ECD covers the binding pocket, reducing the distance of ECD to ECL2, ECL3, as well as ECL1 and transmembrane segments (Figure 1B), thereby the embedding of ECD substantially influences the signaling potency.

### 2.2. Structural Dynamics of GLP-1R for Ligand-Bound and Ligand-Free Conditions

**Figure 2:**
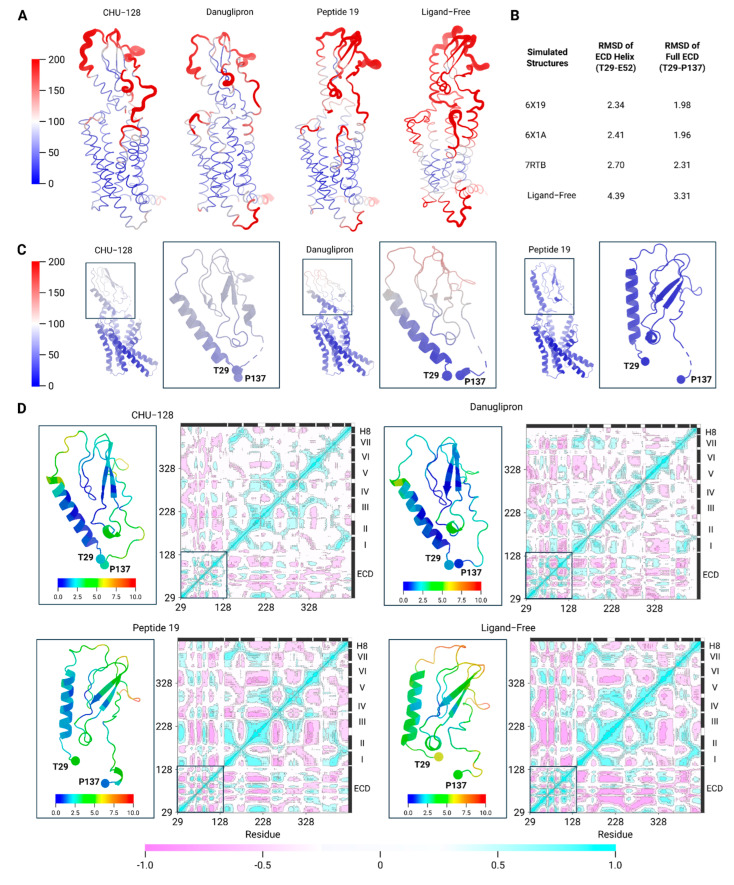
Residue-level dynamic cross-correlation maps of GLP-R during MD simulations. (**A**) Visualization of Cα backbone atom displacement in the ECD region, where blue and red represent low and high fluctuations. Representation of the receptor conformational motion in putty representation. The receptor structure is colored by B-factor values of GLP-1R estimated from the fluctuations of residues during simulation. (**B**) RMSD of ensemble simulation structures of GLP-1R-ligand complexes. (**C**) Representation of experimental structures of ECD region colored by the experimentally determined B-factor. (**D**) The dynamic cross-correlation analysis reveals correlated motions among key structural domains of the receptor, including ECD, ECL, ICL, and TM regions. The correlation coefficients range from −1 to 1, representing the complete negative correlation (blue) and complete positive correlation (red), respectively. The sequence of GLP-1R is color-coded by domain: ECD (yellow), ECL (blue), ICL (green), TM (pink), and Helix 8 (purple). Each simulation was run for 500 ns with *n* = 3 independent replicates. The Roman numbers from I to VII indicate TM segments of the receptor.

To check how stable each structure is, we performed all-atom MD simulations and summarized the systems in Table 2. In the simulations of the ligand–receptor complexes, the receptor backbone and most the ligand conformations remained stable throughout the trajectory (Figure 2A and Figure S1), suggesting equilibrium binding for most of the simulated ligands. However, the danuglipron-bound complex exhibited notable conformational deviation of the ligand after 200 ns simulations (Figures S1 and S2). Different from other compounds, the danuglipron-bound structure did not preserve a thermodynamic stability in the binding pocket, in agreement with prior reports [30] (Figure 2A,C). This observation suggests that danuglipron maintains a relative flexible conformation, implying a faster dissociation rate.

In contrast, the ligand-free simulation revealed much larger receptor structural variations. In the absence of the peptide stabilization, the receptor showed a dramatically increased conformational distribution, especially in the ECD region (Figure 2A,B and Figure S3). Unlike the stable conformation observed in replica 2, a larger conformational motion in replicas 1 and 3 cause the longitudinal ECD structure to cover upon the binding pocket, imitating that of a nonpeptide-bound state. Furthermore, the residual fluctuation plot revealed that increased fluctuations are typically associated with solvent-exposed regions, including the extracellular and intracellular loops and the ECD (Figure 2A,D and Figure S1). Compared to the ligand-free state, the average fluctuation in the ECD is attenuated in the presence of bound ligand, suggesting that ligand engagement stabilizes this domain. These findings imply that the receptor performs transitions to an intermediate, partially active conformation upon ligand removal.

In addition, the dynamic cross-correlation (DCC) analysis further illustrates residue-residue correlation during simulations (Figure 2D). In agonist-bound structures, the ECD exhibits strong positive correlations to ECL1 and ECL2, which are associated with selective downstream signaling. In the danuglipron-bound structure, additional correlation was observed between the ECD and the extracellular portion of TM7. This feature is absent in CHU-128- but present in Peptide 19-bound structures, highlighting potential involvement of TM1-TM7 coupling in regulating G protein-independent signaling [41]. Interestingly, correlation between TM1-TM2 and TM5-TM6 was evident in danuglipron- and Peptide 19-bound systems but not in CHU-128. This intramolecular interaction network may underlie balanced G protein/β-arrestin signaling. In contrast, CHU-128 maintains structural flexibility within G protein signaling-associated segments. In the case of ligand-free structure, the ECD underwent pronounced rearrangement and showed minimal correlation to other domains, instead adopting a more energetically favorable conformation over the simulation time (Figure S3). Furthermore, we observed enhanced correlated motions among ECL3, ICL3, TM5, and TM6, regions essential for signal transduction [42]. While the holo state exhibited more localized, direct residue–residue contacts within the transmembrane helices. The ligand-free state demonstrated overall increased flexibility and dynamic coupling, probably due to the absence of both the ligand and the G protein.

### 2.3. Structural Plasticity of GLP-1R in the Holo State

**Figure 3:**
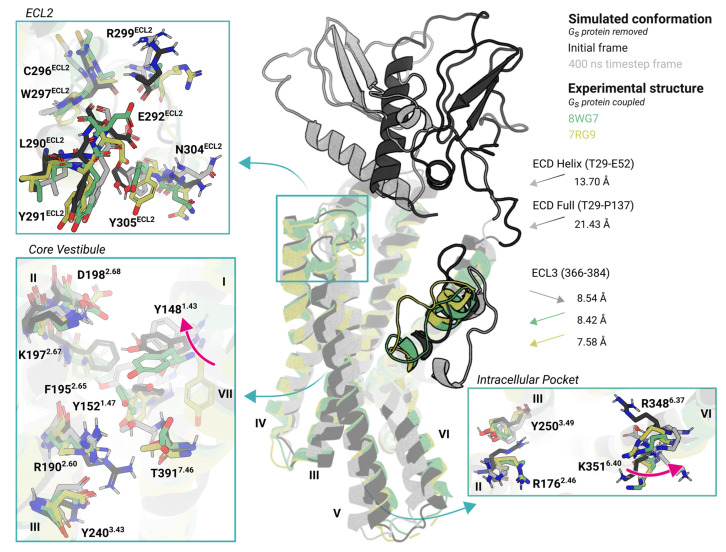
Structural superimposition of ligand-free conformations of GLP-1R. Experimental (yellow: PDB ID 7RG9; green: PDB ID 8WG7) and simulated ligand-free structures (black: initial simulation frame; gray: last simulation frame) are aligned. The superimposed models highlight conformational transitions, particularly in ECL2, the G protein binding pocket, and the core vestibule. Key residue orientations in the simulated structures show notable convergence toward experimentally observed conformations. The RMSDs were computed between the C_α_ atoms of corresponding residues of the two structures, revealing substantial structural variability in the ECD and ECL3 regions. Each simulation was run for 500 ns with *n* = 3 independent replicates. The Roman numbers from I to VII indicate TM segments of the receptor. The gray, green, and yellow arrows represent the move of regions compared to the initial ligand-free simulated structure. The pink arrows in the enlarged box views indicate fluctuations of residues.

To further establish the inherent dynamics of the receptor binding, we compared the simulated ligand-free structures to experimentally resolved ligand-free structures in complex with G proteins. Although these experimental structures have the capacity to engage in downstream signaling, the activation process shows reduced efficacy and frequency [32]. Our simulations captured near-native conformations of ligand-free structures (Figure 3), but two regions exhibited noticeable differences: the ECD and TM6-ECL3-TM7 segment. In the reference structure (initial frame), the helical structure of the ECD is oriented parallel to the transmembrane domain; however, in two simulation replicas, it is repositioned deeper in the orthosteric binding pocket (Figure S3). Notably, the ECD helix undergoes a pronounced shift of approximately 18 Å toward the ECL1 and ECL2 interface (Figure 3), a transitional conformation engaged that is rarely observed in computational approaches [43] or experimental structures [33]. This repositioning may result in transient ECD transmembrane contacts that influence the receptor’s conformational ensemble. Nevertheless, the resulting structure appears insufficiently stabilized to achieve full activation and remains less stable than agonist-bound conformations [33].

### 2.4. Binding Pocket Variants upon Danuglipron, CHU-128, and Peptide 19 Binding

**Figure 4:**
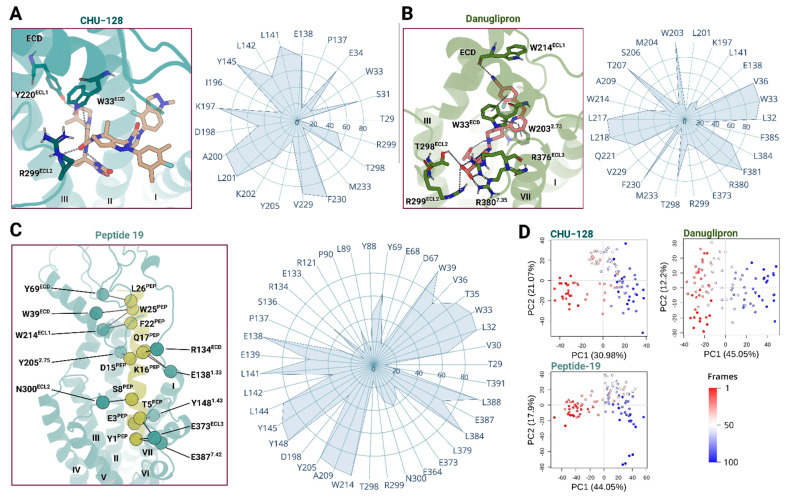
Distinct binding modes of GLP-1R agonists. Distinct properties of the binding pocket of GLP-1R in complex with three agonists obtained from MD simulations: (**A**) CHU-128 (blue: receptor; beige: ligand), (**B**) danuglipron (green: receptor; red: ligand), (**C**) Peptide 19 (cyan: receptor; yellow: ligand). Each ligand adopts a unique binding pose within the orthosteric site of the receptor. The receptor–ligand contact networks remain stable throughout the MD simulations, indicating sustained interactions that support their distinct pharmacological profiles. (**D**) The principal component of residues contribution in structural motions. PC1 captures 31%, 45%, and 44% of total variance in trajectory conformation set of CHU-128-, danuglipron-, and Peptide 19-bound complexes, respectively. Conformations are colored from red to blue in order of simulation time. The Roman numbers from I to VII indicate TM segments of the receptor.

In the presence of a ligand, the ECD stabilizes the orthosteric binding pocket and forms multiple interactions with the ligand throughout the simulations. In small molecule-bound structures, residue W33^ECD^ engages in π-stacking interactions with the indole group of CHU-128 and pyridine ring of danuglipron along the simulations. In addition, both ligands form hydrogen bonds with R299^ECL2^, anchoring the ligand to extracellular loop 2. Despite these shared interactions, CHU-128 and danuglipron exhibit distinct binding features at the ECL3 and TM7 interface (Figure 4A,B). The acid carboxylic group of danuglipron forms polar interactions with ECL3 (R376^ECL3^) and TM7 (R380^7.35^), reinforcing a closed conformation of the TM6-ECL3-TM7 region. However, the dimethylfluorobenzene group of CHU-128 creates a hydrophobic environment that pushes polar residues of TM6-ECL3-TM7 away, thereby expanding the binding cavity.

Intriguingly, Peptide 19 also interacts with TM7 and ECL2 but still induces an opened binding pocket conformation (Figure 4C). This difference arises because Peptide 19 is engaged in extensive interactions with the receptor core, effectively acting as a semi-transmembrane helix during activation. In agreement with previous reports [39], Peptide 19 is stabilized through sustained interactions between the C-terminal region of the peptide and the ECD. Although Peptide 19 shares high sequence similarity with endogenous GLP-1, a tyrosine in Peptide 19 instead of histidine at the N-terminal position (residue 1) introduces steric hindrance with Q234^3.33^, redirecting its orientation toward TM5. The tyrosine transiently interacts with R310^5.44^, disrupting the contact with the ECL3 region and liberating the TM6-ECL3-TM7. During the simulations, the peptide maintained stable electrostatic interactions with E387^7.42^, limiting the degree of TM7 displacement observed in ligand-free structure simulations. These interactions contribute to the peptide’s consistent positioning within the orthosteric site and its ability to regulate transmembrane packing, supporting both G protein-dependent and -independent signaling pathways.

### 2.5. Signal Transduction Associated with Distinct Types of Ligand Binding

#### 2.5.1. Ligand Recognition and Binding via the Extracellular Domain

**Figure 5:**
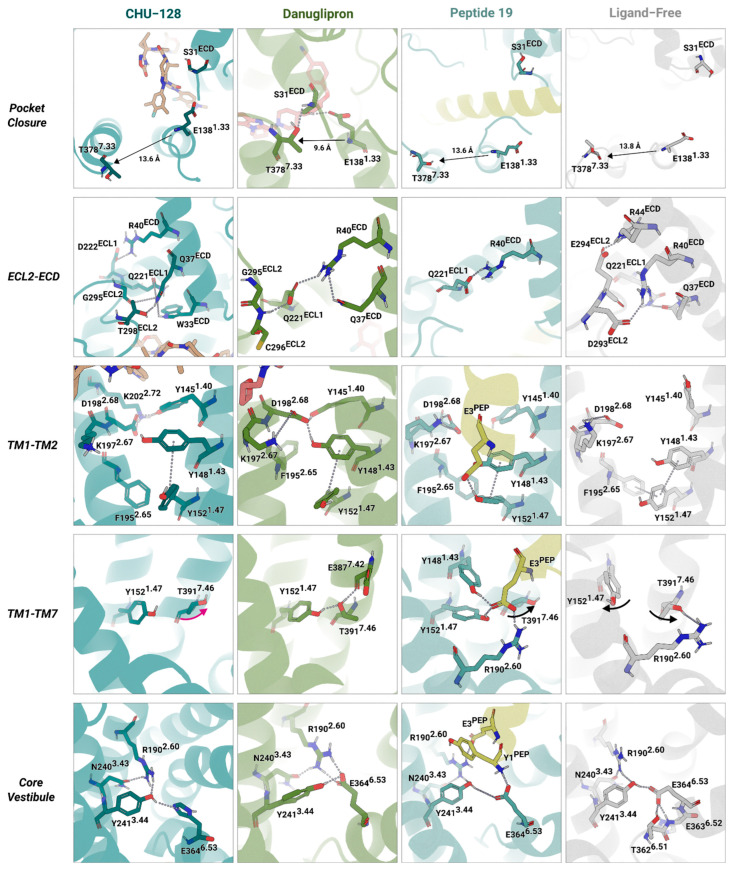
Residue interaction networks of GLP-1R at different ligand binding conditions. Structural configuration of GLP-1R bound to CHU-128 (blue: receptor; orange: ligand), danuglipron (green: receptor; pink: ligand), Peptide 19 (cyan: receptor; yellow: ligand), and ligand-free (gray: receptor) states are shown. The packing of the receptor is intrinsically mediated by the specific agonist. Danuglipron requires simultaneous engagement of the TM6–ECL3–TM7 region to mimic the signal transduction observed in peptide agonists. Peptide 19 also involves this region in receptor activation. In contrast, CHU-128-bound and ligand-free structures exhibit a less compact arrangement of the seven transmembrane helices. Each simulation was run for 500 ns with *n* = 3 independent replicates.

Previous studies have emphasized the important role of W33^ECD^ for ligand recognition and binding [29,44] (Figure 4). However, the specific chemical architecture of each ligand induces distinct extracellular rearrangements, which are followed by conformational changes in TM1 and TM7 (Figure 5). The binding mode of danuglipron promotes a compact conformation by stabilizing the ECD toward TM1 and TM7, leading S31^ECD^ to form a hydrogen bond network with E138^1.33^ and T378^7.33^, thereby repositioning TM7 closer to TM1 and effectively enclosing the binding pocket. In contrast, the ligands CHU-128 and Peptide 19 hinder this convergence by acting as steric barriers. This results in an increased distance between E138^1.33^ and T378^7.33^ (~13.6 Å of C_α_ of residues), preventing closure of the TM1-TM7 interface.

CHU-128 displaces the ECD toward ECL2, forming strong and stable polar interaction with G295^ECL2^ and T298^ECL2^ (Figure 5), while danuglipron and Peptide 19 show either very transient or no hydrogen bonds at this interface. Stabilization of the ECD is further reached by Q221^ECL1^, which forms persistent hydrogen bonds with Q37^ECD^ and R40^ECD^ (Figure 5). Interestingly, ECL1 interacts preferentially with Q37^ECD^ over R40^ECD^ in CHU-128-bound and ligand-free structures but favors interaction of R40^ECD^ with ECL1 in danuglipron- and Peptide 19-bound structures. This may be associated with balanced agonists that pull away the ECD from ECL1-ECL2; thus, only the longer side chain of R40^ECD^ maintains the contact with ECL1. Overall, these ligand–receptor interaction patterns define the orientation and spatial dynamics of the ECD in the binding pocket during MD simulations. Notably, the binding mode of CHU-128 and danuglipron are typical for G protein-biased and balanced nonpeptide agonists, respectively, inducing the ECD toward either ECL2 or TM1-TM7 regions. Despite CHU-128 inducing a more open binding pocket, which intuitively is associated with rapid ligand dissociation, cell-based experiments report a higher binding affinity for CHU-128 compared to danuglipron [30]. Thus, the dissociation kinetics may not only be governed by transmembrane rearrangements but also by the hydrophobic character of the ligand, which affects retention within the solvent-exposed binding pocket. Danuglipron, by enclosing the binding pocket, gains increased binding energy to stabilize structure packing. As class B GPCRs typically accommodate large peptide ligands via expanded orthosteric binding pockets, the evolutionary design includes an extended N-terminal domain to realize high-affinity binding [45].

#### 2.5.2. Signal Transduction Along the Transmembrane Domain

Within the core vestibule, key residues in TM1, TM2, TM3, and TM7 contribute to helical packing. In the ligand-free simulated structures, a transient π-stacking network forms among Y148^1.43^, Y152^1.47^, and F195^2.58^. This network occasionally extends to Y145^1.40^, K197^2.67^, D198^2.68^, and K202^2.72^ in the holo state structure (Figure 5), enhancing TM1 packing with other TM regions. However, in ligand-free structures, Y145^1.40^ shift outward, disrupting the interaction with TM1-TM2 contact and leading to expansion of the TM6-ECL3-TM7 interface, enlarging the open conformation. These rearrangements lead to the greater dynamics within the TM helical bundle and extends the binding pocket. In contrast, ligand-bound structures exhibit reduced transmembrane flexibility, stabilizing an active-like conformation. In Peptide 19-bound structures, Y145^1.40^ interacts with K202^2.72^ mediated by Y10^PEP^, reinforcing TM1-TM2 association (Figure 4). For small molecules, the receptor forms consistently direct interactions between D198^2.68^ with Y145^1.40^ and Y148^1.43^ in danuglipron-bound structures and supplementary interactions K202^2.72^ and Y145^1.40^ for CHU-128-bound structures, but the functional role of Y148^1.43^ and Y152^1.47^ differ in the core vestibule. Specifically, in danuglipron-bound structures, Y152^1.47^ forms hydrogen bonds with T391^7.46^, pulling TM1 toward TM7 and priming the receptor for β-arrestin-dependent signaling. In case of Peptide 19, the peptide bridges TM1 and TM7, stabilizing the core vestibule and supporting both G protein- and β-arrestin-dependent pathways. In contrast, the simulations of CHU-128-bound and ligand-free structures exhibit disengagement of Y152^1.47^ from TM7, implying that these configurations are less favorable TM helical packing. Furthermore, Y152^1.47^ often shifts from the core to the membrane periphery, suggesting a functional role during activation.

**Figure 6:**
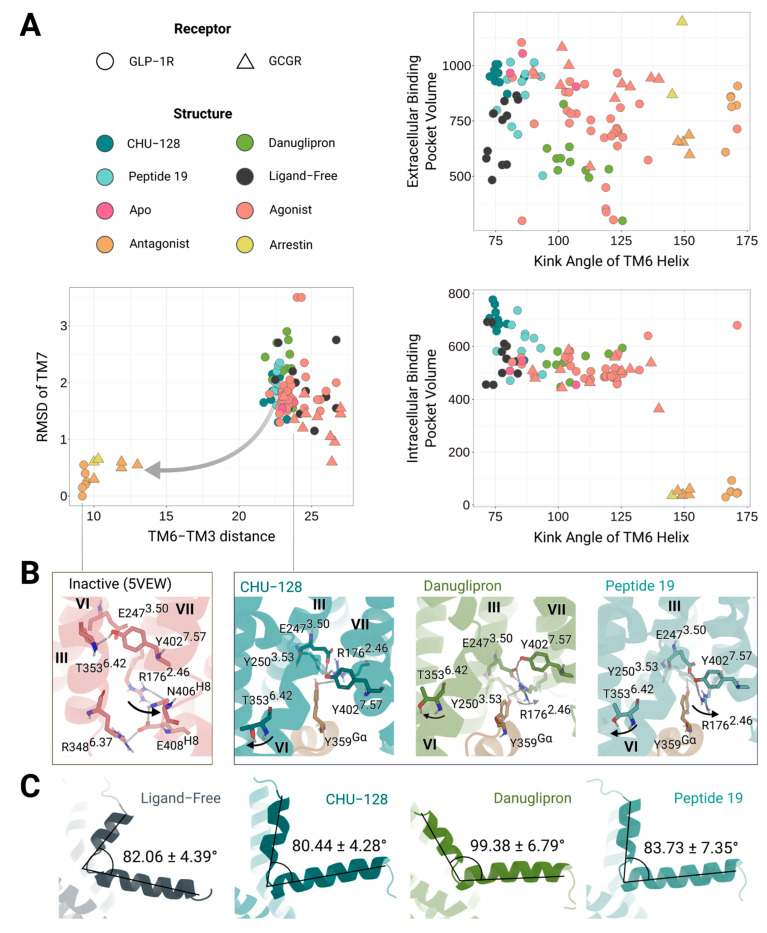
Conformational adaption of the intracellular signaling pocket of GLP-1R upon binding of different ligands. (**A**) Structural comparison between the G protein-bound active state and the inactive state of GLP-1R. The RMSD of TM7 (Å) is measured using C_α_ atoms of residues 7.49 and 7.54, with reference to the inactive state structure (5VEW). The distance (Å) between TM3 and TM6 is measured between residues 3.50 and 6.34. The volumes of the intracellular and extracellular binding sites (Å^3^) are calculated with POVME. (**B**) Intramolecular interactions of the core vestibule. Different residues are involved in the structural states through rotation and kinking of TM6. (**C**) Structural representation of TM6 kink. The angle was calculated from residue I345, G361, and F367. The roman number III, VI to VII indicates the TM3, TM6, and TM7 of receptor.

In addition, TM6 is integrally involved in the TM helical packing. An approximate 90° kink of TM6 is a hallmark of class B GPCR activation, distinguishing it from class A GPCRs. In our simulations, the kink was present in the active and in the ligand-free state, unless external stimuli forced TM6 to engage a less kinked conformation. In all simulated structures, we observe that ligand-free structures exhibit the largest opening, where the kink angle deviation is comparable to that of CHU-128-bound structures (Figure 6). This suggests that the TM6 kink is adjusted to optimally bind large peptide ligands that modulate the downstream signaling. In the basal state, the TM6 conformation is preferentially shaped to the association of the G protein. Binding of danuglipron induced a straighter TM6 helix conformation, with kink angles fluctuating between 100 and 125°, still approximately 20–30° greater than those found in CHU-128-bound structures (Figure 6). This conformational bias is supported by strong polar interactions across the binding pocket, such as between E34^ECD^-T378^7.33^, R299^ECL2^-R380^7.45^, R299^ECL2^-E373ECL3, R3105.44-E373ECL3, S301ECL2-E376ECL3, and R1902.60-E3646.53, which constrain TM6 straightening (Figure 5 and Figure S3).

#### 2.5.3. Features of the Intracellular Binding Domain

To stabilize the active state, residue Y402^7.53^ interacts with R176^2.39^, E247^3.46^, and T355^6.39^, enhancing stability further by engaging Y359 of the G protein (Figure 6B and Figure S5). R176^2.46^ performs a transition from an open to a closed locked state through a shift toward the H8 and the Y402^7.53^ interacts with T355^2.48^, maintaining the TM6 in a kinked conformation. During the deactivation process, the TM6 undergoes exploratory shifts and rotations toward the intracellular binding site, releasing Y402^7.53^, which then orients toward the core vestibule, forming polar interaction with R247^3.50^ and T353^6.37^ (Figure 6A). Residue R176^2.46^ further reinforces the closed conformation by forming a hydrogen bond network with N406^8.46^, E408^8.48^, and R348^6.37^, thereby firmly closing the structure. In the active state, residues N406^8.46^ and E408^8.48^ form transient interaction with the G protein, and E408^8.48^ is believed to initiate G protein binding [46]. In the absence of the G protein, residue N406^8.48^ forms an internal interaction with the backbone of Y402^7.53^, suggesting that the N-terminal of H8 plays a critical role in recognizing G protein and maintaining the structural stability.

Structural analysis of the intracellular binding pocket reveals a variation in the binding mode of the G protein when danuglipron is bound to the receptor, as compared to other receptor–ligand complexes (Figure 6B and Figure S6). During simulation, the G protein in the danuglipron-bound structure shifts closer to the TM3-TM5-TM6 regions, resulting in detachment from ICL2 and H8. This movement suggests that danuglipron induces a distinct conformation of the receptor, which is less stable or less tightly associated with the G protein than other ligands. In contrast, CHU-128- and Peptide 19-bound structures maintain G protein interactions with both the receptor’s TM3-TM5-TM6 regions and the intracellular loop (ICL2 and H8), likely stabilizing the receptor–G protein complex more effectively and for a longer duration. This distinct variation in the binding mode may explain why danuglipron exhibits lower activity potency than CHU-128 [34], despite both ligands interacting with the receptor and initiating G protein signaling. Thus, differences in ligand binding modes likely influence the efficacy and potency of G protein activation.

Unlike class A GPCRs, GLP-1R lacks the conserved NPxxY^7.53^ motif at the intracellular region of TM7, which is crucial for ligand-induced conformational changes [47]. In class A GPCRs, the asparagine in this motif stabilizes the active/inactive state and is tightly involved in receptor signaling. However, in GLP-1R, the positions 7.49 and 7.50 contain small hydrophobic residues. Furthermore, GLP-1R does possess the conserved Y402^7.53^ residue, which forms direct T-shaped interactions with the tyrosine at the C-terminal end of G protein. These observations suggest that the 7.49 and 7.50 positions play a less important role in receptor activation, implying that peptide hormone ligand-induced receptors may have a distinct activation mechanism [48].

### 2.6. Biased Agonism and Receptor Signaling Mechanisms

#### 2.6.1. Inferring β-Arrestin-Bound to GLP-1R via Structural Comparison with Related Class B1 GPCRs

Interestingly, experimental structures of ligand-free GLP-1R maintain an open conformation of the intracellular binding region (Figure 6A), denoting the receptor to adopt G protein-preferred conformation [33]. The extracellular portions of TM1, TM6, and TM7 shift outward in the ligand-free state in contrast to the inward movements observed upon peptide binding. The CHU-128-bound and ligand-free GLP-1R structures exhibit similar conformations of the receptor in the extracellular regions, preferentially activating the cAMP accumulation pathway. In this context, the packing between TM1, TM2, and TM7 at the intracellular side is critical for β-arrestin recognition and binding (Figure S4). Interestingly, simulation of danuglipron- and Peptide 19-bound receptor complexes revealed that R176^2.46^ rotates toward the H8 of GLP-1R, forming strong polar interaction in inactive receptor state (Figure 6B). We speculate that the rotation of the arginine is a key factor for the transition of the G protein-preferred to the β-arrestin-preferred conformation. Other β-arrestin-bound structures provide additional clarification into the biased signaling mechanism of GLP-1R. The potential conformation of the GLP-1R–β-arrestin complex was deduced from the experimental structure of another glucagon receptor (GCGR)–β-arrestin complex, because GCGR is one of the few classes B1 GPCRs for which structures in complex with β-arrestin are available. In the structures of GCGR–β-arrestin complexes in either glucagon-bound or ligand-free states, TM5 and TM6 exhibit a similar arrangement to that found in the inactive state of GLP-1R (Figure 6A and Figure S4). This suggests that GLP-1R potentially “dekinks” TM6 during β-arrestin binding (Figure S4). Furthermore, the arginine (2.47) interacts with E^8.49^, limiting the intracellular binding pocket to interact with the G protein (Figure 6A). Consequently, balanced agonists compact the overall TM helical bundle structure by shifting TM5, TM6, and TM7 inward, which means that intermediate states are transient ensembles capable of transitioning from active-like to inactive-like conformations.

Although the ligand-free structure allows for the receptor to explore a broader conformational landscape, unbiased MD simulations have to reach full transition from a G protein-adapted conformation to a β-arrestin-adapted state, where the receptor must adopt an inactive-like conformation that promotes β-arrestin binding (Figure S4). The simulated ligand-free structures remain poised to engage G_s_ protein binding, due to the difficulty in reaching the dekinked, straight TM6. In contrast, the ligand-free experimental structures display higher degree of expansion, leading to reduced packing between TM1, TM6, and TM7 to the rest of the structure, and thereby promoting a more frequently opened conformation in the intracellular binding pocket. During the simulations, the hydrogen bonding between R190^2.53^ and T391^7.41^ is disrupted due to the outward displacement of TM7, accompanied by outward shifts in Y148^1.43^ and D198^2.68^ (Figure 3). These results support that GLP-1R can achieve basal activation of cAMP signaling pathways in the absence of bound ligand, while G protein-independent pathways may require further structural stabilization. The unoccupied intracellular region also permits transient side-chain rearrangements that may interfere with stable effector binding.

#### 2.6.2. Promising Pharmacokinetics of Biased Agonists

Balanced agonists typically induce a receptor conformation similar to that of the endogenous GLP-1-bound structure, prerequisite for activation of both G protein and β-arrestin signaling pathways. In contrast, G protein-biased small-molecule agonists, binding at the extracellular interface, promote receptor conformations that preferentially activate G protein signaling while attenuating β-arrestin recruitment [30]. Similar to CHU-128-bound structures, exendin-P5, a G protein-biased ligand, primarily interacts with the TM4-TM5 regions and to lesser extent with TM1-TM7, supporting the hypothesis that signaling pathways are tightly linked to ligand binding orientation within the transmembrane regions [49]. Thus, G protein-biased agonists can disrupt interactions between TM1-TM7, which are associated with downstream protein coupling [50], thereby attenuating β-arrestin engagement.

G protein-biased GLP-1R full agonists, such as P5 and PX17, have been shown to improve glucose control and facilitate weight loss without significant appetite suppression or nausea, which could be attributed to alternative signaling pathways [18,51]. These agonists enhance anti-hyperglycemic efficacy by limiting the structural changes of TM6-ECL3-TM7 and TM1 in GLP-1R, reducing receptor desensitization and internalization [18,30,52]. The binding of CHU-128 induces a significant rearrangement within the binding pocket of GLP-1R and creates a large opening in ECL3, which leads to the weak packing of TM5-TM6. In contrast, danuglipron stabilizes the binding pocket by pulling ECL3 closer to the binding pocket, which unexpectedly attenuates the activity potency of the ligand. Biased agonists that induce prolonged activation enhance the hypoglycemic effect and reducing side effects from the β-arrestin signaling pathway. Another promising approach to enhance the therapeutic curation of metabolic disease is the development of multi-target agonists, such as Peptide 19 and tirzepatide, which improve T2D treatment by combining the therapeutic effects of both GLP-1 and GIP receptors.

## 3. Materials and Methods

### 3.1. Complex Structure Preparation

To explore the structural dynamics of ligand-induced activation of the GLP-1R, we selected three ligand–receptor–G_s_ complexes, comprising danuglipron (PDB ID: 6X1A), CHU-128 (PDB ID: 6X19), or Peptide 19 (PDB ID: 7RTB) as ligands, and the G_s_ protein. Structurally unresolved residues, typically found in extracellular domain (ECD)-TM1 linker and extracellular loop 3 (ECL3) regions, were modeled using the Modeller 10.6 [53] and optimized with the Prepwizard module from the Schrodinger software suite [54]. To simulate the presence of G protein without overburdening the system, only the G_α_ subunit was retained in the complex. For the MD simulation of the receptor alone, we remove the Peptide 19 and the G protein from the published structure of the ternary complex [31].

### 3.2. Molecular Dynamics Simulation Protocol

MD simulations were performed in a membrane-mimicking lipid bilayer system [55,56,57]. The lipid bilayer membrane was established using the CHARMM-GUI webserver [57] and distinct receptor complex structures were integrated in the lipid bilayer using the OPM webserver [58]. The lipid bilayer was composed of 1-palmitoyl-2-oleoylphosphatidylcholine (POPC). The receptor complex structure was embedded in a lipid bilayer of 240 lipids, hydrated to TIP3P water molecules with 0.15 M NaCl. All systems were simulated in GROMACS using CHARMM36m force field [59]. The systems were minimized with a 5000-step energy minimization using the steepest descent algorithm and subjected to temperature equilibrating in the NPT ensemble at 310 K for 500 ps. For objective analysis, simulations were repeated three times with different initial velocities. In all holo-state structures used for simulations, the experimental structures of the G proteins and ligands were maintained, with additional restraints applied to the G protein backbone. For the ligand-free receptor structure, no structural restraints were applied. All replicas underwent 500 ns simulations to visualize large-scale motions. All molecular dynamics simulations were performed using an Ubuntu 22.04 LTS system. The openSUSE Leap 15.1 (Supermicro, Shenzhen, China) was equipped with a 104-core Intel^®^ Xeon^®^ Platinum 8269CY CPU, 245 GB of RAM, and an NVIDIA GeForce RTX 3080 GPU comprising 24 GB VRAM.

### 3.3. Analysis of Simulated Structures

The MD data were analyzed using several methods to characterize the dynamic behavior of the receptor. Root mean square deviation (RMSD) and root mean square fluctuation (RMSF) were computed using the GROMACS 2022.2 [59]. RMSD quantifies the deviation between the reference structure and the molecular trajectory, with the initial frame structure in the simulation typically used as the reference. RMSF evaluates the flexibility of individual atoms or residues throughout the simulation.

The DSSP method was employed to analyze hydrogen bonding patterns and the spatial arrangement of backbone atoms in a protein structure, evaluating the angles between backbone atoms (C_α_-N-C_α_ and N-C_α_-C) and the distance between hydrogen bond donors and acceptors. Based on these hydrogen bonding patterns, DSSP classifies the protein secondary structures into four types including alpha helix, beta strand, turn, and loop [60].

The dynamic cross-correlation (DCC) method, implemented by Bio3D [61], was used to analyze coordinated movement between atoms in the MD trajectory. DCC provides insights into the collective behavior of residues in macromolecules, helping to identify functional dynamics and how different parts of the molecule move relative to one another. In MD simulations, atoms within a molecule continuously move, and DCC quantifies how these atomic displacements correlate over time. The DCC calculation is performed by computing the cross-correlation of atomic displacements over time.

Residue Interaction Network Generator (RING) 4.0 [62] provides an advanced method for detecting and analyzing residue interactions, representing these interactions as a network with residues as nodes and their interaction information as edges, including the interaction type and interaction frequency.

Finally, the binding pocket volume was calculated using the POVME v3.0 tool [63], which characterizes the shape and flexibility of binding pockets within macromolecules. The software uses a cavity detection algorithm to evaluate the 3D spatial arrangement of atoms, identifying potential binding pockets based on their shape, size, and ligand accessibility. The center of the binding pocket was defined as the center of mass of the ligand for ligand-bound structures.

## 4. Conclusions

In summary, this work provides detailed molecular insights into different types of agonists coupling to GLP-1R. We used structure-based MD simulations to resolve G protein-biased and balanced agonist-induced signaling for GLP-1R. The G protein-biased compound CHU-128 stabilizes the receptor structure in active state by maintaining large open conformation of the receptor’s ligand binding pocket. In contrast, the binding of danuglipron assembles extracellular portions of the transmembrane helices, which may require a higher energy barrier to maintain closed conformation of the binding pocket [64]. Notably, a change in the kink angle of 20–30° in the TM6 helix that shifts the extracellular region of TM6 toward the binding pocket is essential to cope with the energy barrier required for prolonged activation [64]. In addition, a limitation of using small-molecule ligands as potential drugs is their inability to simultaneously modulate the extracellular domain, the extracellular parts of transmembrane domains, and the core TM vestibule. However, they can still mimic the activity of peptide agonists, offering alternative assets to enhance the oral bioavailability and metabolic stability of GLP-1R-targeting drugs. Our MD simulations showed that balanced agonist-bound structures tend to adapt a G protein-independent signaling conformation in the intracellular binding region. As class B receptors, GLP-1R can adopt similar inactive-like conformation as found for GCGR to engage β-arrestin but may not reach the full β-arrestin active state of the receptor in the absence of particular agonists.

## Figures and Tables

**Table 1:** Information of simulated ligands. The residue positions for the peptide sequence are numbered at position 1 (N-terminus) and at position 38 (C-terminus).

Compounds	Danuglipron	CHU-128	Peptide 19
Chemical structure	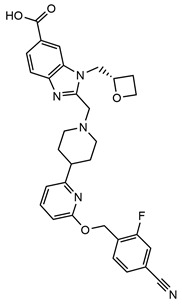	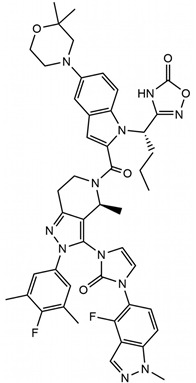	Y^1^AEGTFTSDYSIY LDKQAAAEFVNW LLAGGPSAPPPSK^38^
Molecular mass	555.6 g/mol	883.96 g/mol	4064 g/mol
Ligand type	Small molecule	Small molecule	38-residue peptide
Activity type	Balanced	G protein-biased	Balanced
Binding site	Orthosteric	Orthosteric	Orthosteric

**Table 2:** Summary of GLP-1R simulated systems.

PDB	Simulation Time	Replicates	Resolution (Å)	Activity State	Ligand	Ligand Type	Downstream Protein
6X1A	500 ns	3	2.50	Active	Danuglipron	Balanced agonist	G_s_
6X19	500 ns	3	2.10	Active	CHU-128	Biased agonist	G_s_
7RTB	500 ns	3	2.14	Active	Peptide 19	Balanced agonist	G_s_
7RTB	500 ns	3	2.14	Ligand-Free	-	-	-

## Data Availability

Data are contained within the article and Supplementary Materials.

## Supplementary Materials

The following supporting information can be downloaded at: https://www.mdpi.com/article/10.3390/molecules30153216/s1, Table S1: List of all GLP-1R structures and inactive and beta-arrestin-bound GCGR structures; Figure S1: Evaluation of the conformational change, stability, and flexibility of GLP-1R in ligand-free and ligand-bound states; Figure S2: Conformational changes at the extracellular binding pocket upon danuglipron binding to GLP-1R; Figure S3: Structural dynamics of ligand-free GLP-1R by MD simulation; Figure S4: Comparison of β-arrestin-bound and inactive state incretin structures; Figure S5: Comparison of biased and balanced agonist-bound GLP-1R structures; Figure S6. Distinct conformational transitions in the intracellular signaling pocket upon extracellular binding of different ligand to GLP-1R.

## Abbreviations

GLP-1R: glucagon-like peptide 1 receptor; SM: small molecule; MD: molecular dynamics; GPCR: G protein-coupled receptor; T2B: type 2 diabetes; ECD: extracellular domain; GLP-1RA: GLP-1R agonist; RMSD: root mean square deviation; RMSF: root mean square fluctuation; DCC: dynamic cross-correlation; RING: Residue Interaction Network Generator; H8: Helix 8; GCGR: glucagon receptor.
