# Supplementary material for: Exploring Conformational Transitions in Biased and Balanced Ligand Binding of GLP-1R
Source: Molecules. 2025 Jul 31;30(15):3216. doi: 10.3390/molecules30153216 (PMC12348053; doi:10.3390/molecules30153216)
Supplement: Supplementary file 1 [file molecules-30-03216-s001.zip › molecules-3729264-supplementary.pdf]

Supporting Information

Exploring Conformational Transitions in Biased and Balanced Ligand Binding of GLP-1R

Marc Xu [1,2], Horst Vogel [1,3,4,*] and Shuguang Yuan [1,5,*]

[1] Research Center for Computer-Aided Drug Discovery, Shenzhen Institutes of Advanced Technology, Chinese Academy of Sciences, Shenzhen 518055, China

[2] University of Chinese Academy of Sciences, Beijing 100049, China

[3] Faculty of Pharmaceutical Sciences, Shenzhen University of Advanced Technology, Shenzhen 518055, China

[4] Institut des Sciences et Ingénierie Chimiques (ISIC), Ecole Polytechnique Fédérale de Lausanne (EPFL), 1015 Lausanne, Switzerland

[5] AlphaMol Science Ltd., Shenzhen 518055, China

* Correspondence: horst.vogel@epfl.ch (H.V.); shuguang.yuan@cadd2drug.org (S.Y.)

Table S1. List of all GLP-1R structures and inactive and beta-arrestin-bound GCGR structures. *The potency of molecule is considered as G protein biased when it cannot or weakly activate the arrestin signaling.* ______ 3

Figure S1. Evaluation of the conformational change, stability, and flexibility of GLP-1R in ligand-free and ligand-bound states. *Ligand–receptor complexes maintain a stable conformation over the course of the simulation, while ligand-free structures exhibit increased instability, notably in the ECD domain. RMSD is computed for both the receptor backbone and the ligand relative to the initial frame and RMSF estimates the flexibility of residues in receptor structure. All the simulations are repeated three times.* 5

Figure S2. Conformational changes at the extracellular binding pocket upon danuglipron binding to GLP-1R. *Specific motion are indicated by arrows. In replica 1, the ligand shifts upward, suggesting the potential dissociation from the receptor. In replica 2, the initial tight binding of ECL2 and ECL3 shows a progressive loss of the interaction network, characterized by an opening of the binding pocket.* 6

Figure S3. Structural dynamics of ligand-free GLP-1R by MD simulation. *The conformation of the ECD adopts distinct shapes across different replica simulations. In replica 1, the ECD is oriented towards ECL2, whereas in replica 3, the ECD tilts forward within the binding pocket.* 7

Figure S4. Comparison of β-arrestin-bound and inactive state incretin structures. *Visualization of the internal interaction network and structural disorder in the extracellular and intracellular binding pockets, as well as the core vestibule. Five structures were selected: 5VEW and 6KK1 represent the inactive state of GLP-1R, 5XEZ is the inactive structure of GCGR, and 8JRU and 8JRV correspond to the β-arrestin-bound structure of GCGR.* ... 8

Figure S5. Comparison of biased and balanced agonist-bound GLP-1R structures. *A) Alignment of different agonist-bound GLP-1R structures. Balanced small molecules induce a closure of the binding pocket. B) Interaction fingerprint of biased and balanced small molecules in the binding pocket. Both types of ligands induce distinct polarity environment in the TM6-ECL3-TM7 region.* 9

Figure S6. Distinct conformational transitions in the intracellular signaling pocket upon extracellular binding of different ligand to GLP-1R. *Visualization of the G protein binding mode in the intracellular G protein binding pocket. During MD simulation of the GLP-1R/danuglipron/G protein complex the G protein lost its interaction with ICL2 and H8 of the receptor.* 10

Table S1. List of all GLP-1R structures and inactive and beta-arrestin-bound glucagon receptor (GCGR) structures. The potency of molecule is considered as G protein-biased when it cannot or weakly activate the arrestin signaling.

PDB	Receptor	State	Binder	Position	Type	Activity	Signaling	Potency
5NX2	GLP-1R	Active	Truncated peptide	Orthosteric	Pep	Agonist	Unknown	
5VAI	GLP-1R	Active	GLP-1	Orthosteric	Pep	Agonist	Balanced	Full
5VEW	GLP-1R	Inactive	PF-06372222	Allosteric	SM	Antagonist		
5VEX	GLP-1R	Inactive	NNC0640	Allosteric	SM	Antagonist		
6B3J	GLP-1R	Active	Exendin-P5	Orthosteric	Pep	Agonist	G protein-biased	Full
6KJV	GLP-1R	Inactive	PF-06372222	Allosteric	SM	Antagonist		
6KK1	GLP-1R	Inactive	PF-06372222	Allosteric	SM	Antagonist		
6KK7	GLP-1R	Inactive	PF-06372222	Allosteric	SM	Antagonist		
6LN2	GLP-1R	Inactive	PF-06372222	Allosteric	SM	Antagonist		
6ORV	GLP-1R	Active	TT-OAD2	Orthosteric	SM	Agonist	G protein-biased	Partial
6X18	GLP-1R	Active	GLP-1	Orthosteric	Pep	Agonist	Balanced	Full
6X19	GLP-1R	Active	CHU-128	Orthosteric	SM	Agonist	G protein-biased	Full
6X1A	GLP-1R	Active	PF-06882961 (danuglipron)	Orthosteric	SM	Agonist	Balanced	Full
6XOX	GLP-1R	Active	OWL-833	Orthosteric	SM	Agonist	G protein-biased	Partial
7C2E	GLP-1R	Active	RGT1383	Orthosteric	SM	Agonist	Balanced	Full
7DUQ	GLP-1R	Active	GLP-1	Orthosteric	Pep	Agonist	Balanced	Full
7DUR	GLP-1R	Active	Compound 2	Allosteric	SM	Agonist	G protein-biased	Full
7E14	GLP-1R	Active	OWL-833	Orthosteric	SM	Agonist	G protein-biased	Partial
7EVM	GLP-1R	Active	Compound 2	Allosteric	SM	Agonist	G protein-biased	Full
7FIM	GLP-1R	Active	Tirzepatide	Orthosteric	Pep	Agonist	G protein-biased	Full
7KI0	GLP-1R	Active	Semaglutide	Orthosteric	Pep	Agonist	Balanced	Full
7KI1	GLP-1R	Active	Taspoglutide	Orthosteric	Pep	Agonist	Balanced	Full
7LCI	GLP-1R	Active	PF-06882961 (danuglipron)	Orthosteric	SM	Agonist	Balanced	Full
7LCJ	GLP-1R	Active	PF-06882961 (danuglipron)	Orthosteric	SM	Agonist	Balanced	Full
7LCK	GLP-1R	Active	PF-06882961 (danuglipron)	Orthosteric	SM	Agonist	Balanced	Full
7LLL	GLP-1R	Active	Exendin-P5	Orthosteric	Pep	Agonist	G protein-biased	Full

7RG9	GLP-1R	Active	-	-	Apo	Apo		
7RGP	GLP-1R	Active	Tirzepatide	Orthosteric	Pep	Agonist	G protein-biased	Full
7RTB	GLP-1R	Active	Peptide 19	Orthosteric	Pep	Agonist	Balanced	Full
7S15	GLP-1R	Active	PF-06883365	Orthosteric	SM	Agonist	Balanced	Full
7S1M	GLP-1R	Active	Ex4-4D-A	Orthosteric	Pep	Agonist	Balanced	Full
7S3I	GLP-1R	Active	-	-	Apo	Apo		
7VBH	GLP-1R	Active	Peptide 20	Orthosteric	Pep	Agonist	G protein-biased	Full
7VBI	GLP-1R	Active	Non-acylated tirzepatide	Orthosteric	Pep	Agonist	Unknown	Full
7X8R	GLP-1R	Active	Boc5	Orthosteric	SM	Agonist	G protein-biased	Full
7X8S	GLP-1R	Active	WB4-24	Orthosteric	SM	Agonist	G protein-biased	Full
8JIP	GLP-1R	Active	MEDI0382	Orthosteric	Pep	Agonist	Balanced	Full
8JIR	GLP-1R	Active	SAR425899	Orthosteric	Pep	Agonist	Balanced	Full
8JIS	GLP-1R	Active	Peptide 15	Orthosteric	Pep	Agonist	Balanced	Full
8WG7	GLP-1R	Active	-	-	Apo	Apo		
8YW3	GLP-1R	Active	Retatrutide	Orthosteric	Pep	Agonist	Unknown	Full
9IVG	GLP-1R	Active	GLP-1(9-36)	Orthosteric	Pep	Agonist	Balanced	Full
9IVM	GLP-1R	Active	GLP-1(9-36)	Orthosteric	Pep	Agonist	Balanced	Full
9J1P	GLP-1R	Active	g1:Ox	Orthosteric	Pep	Agonist	Balanced	Full
8JRU	GCGR	Active	-	-	Apo	Apo		
8JRV	GCGR	Active	GLP-1	Orthosteric	Pep	Agonist	Balanced	Full
5XF1	GCGR	Inactive	NNC0640	Allosteric	SM	Antagonist		
5XEZ	GCGR	Inactive	NNC0640	Allosteric	SM	Antagonist		
5EE7	GCGR	Inactive	MK-0893	Orthosteric	SM	Antagonist		
4L6R	GCGR	Inactive	Diethylene glycol	Allosteric	SM	Antagonist		

Pep: Peptide; SM: Small molecule

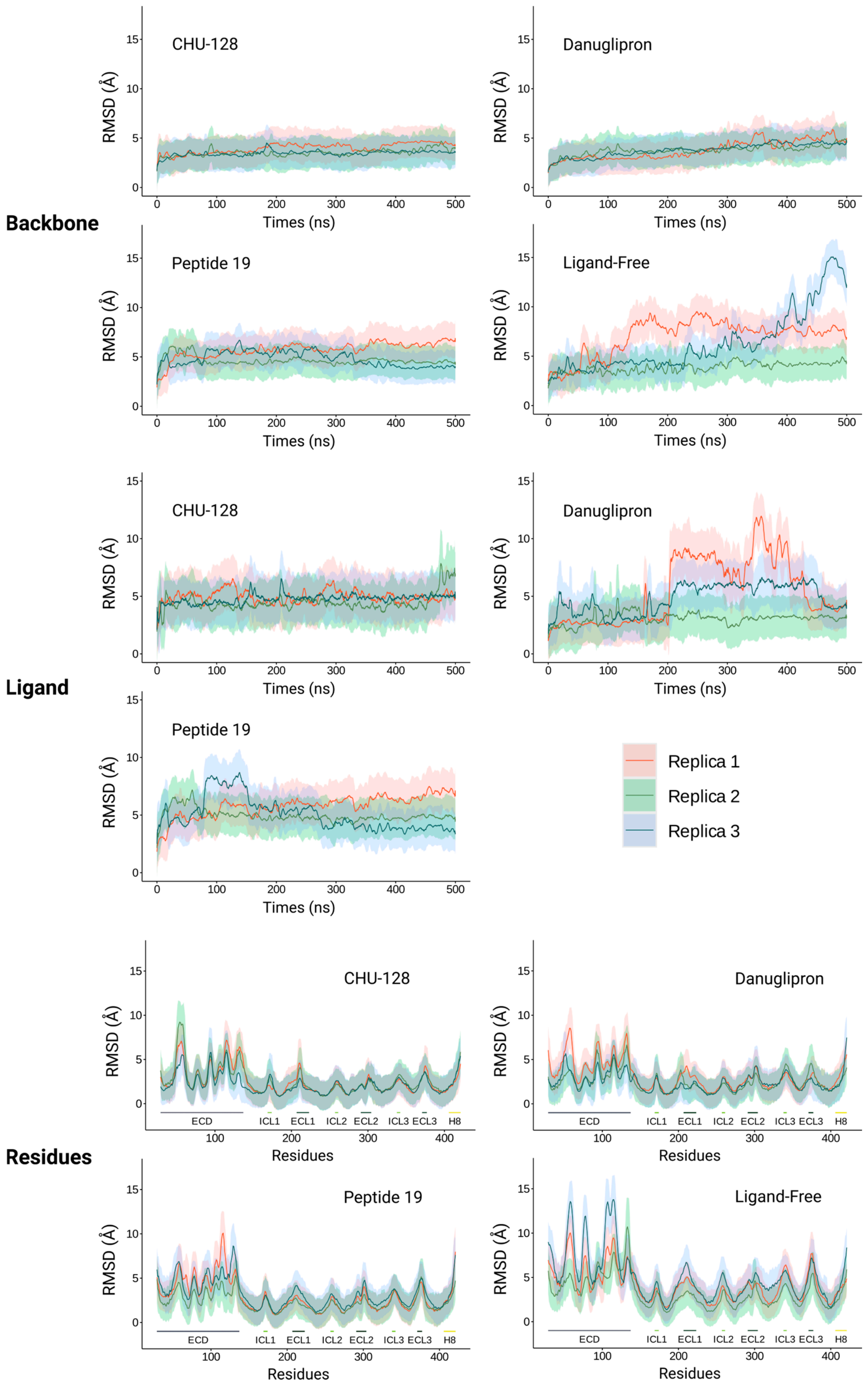

Figure S1. Evaluation of the conformational change, stability, and flexibility of GLP-1R in ligand-free and ligand-bound states. Ligand–receptor complexes maintain a stable conformation over the course of the simulation, while ligand-free structures exhibit increased instability, notably in the ECD domain. RMSD is computed for both the receptor backbone and the ligand relative to the initial frame and RMSF estimates the flexibility of residues in receptor structure. All simulations are repeated three times.

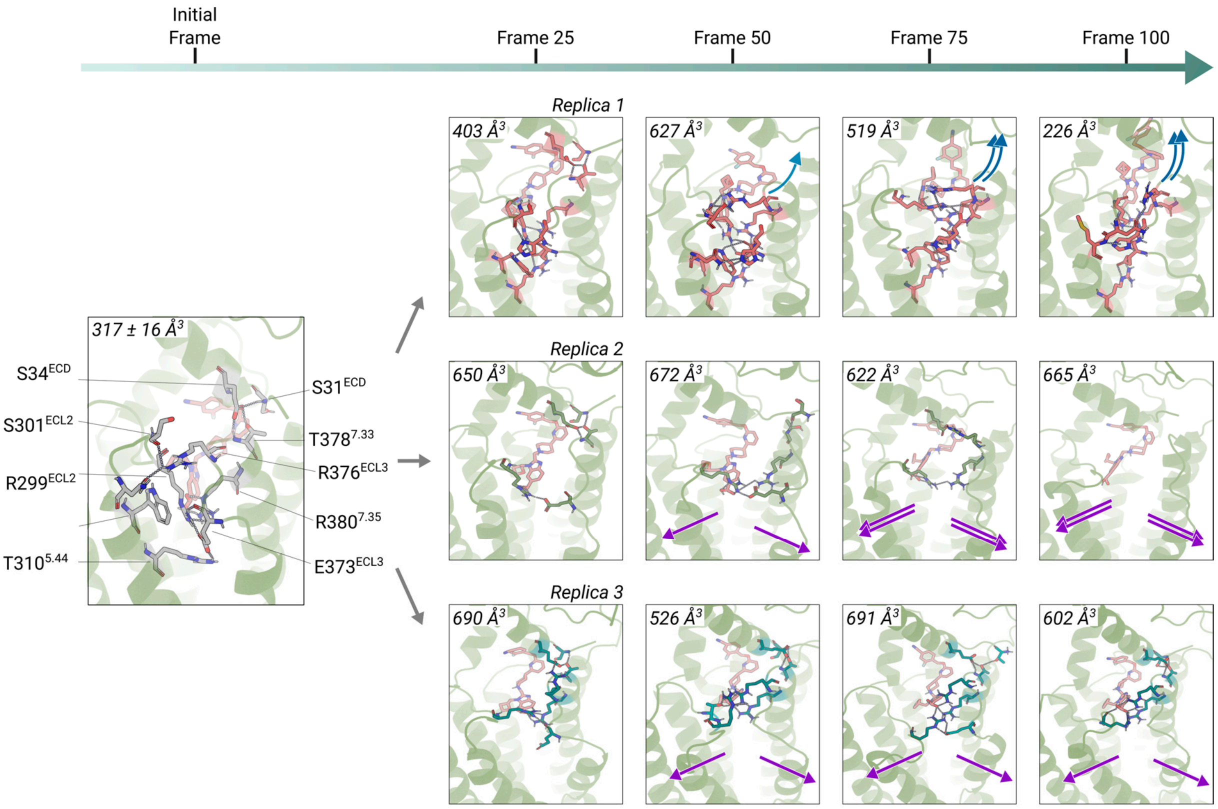

Figure S2. Conformational changes at the extracellular binding pocket upon danuglipron binding to GLP-1R. The computed volume represents the spatial volume of the binding pocket calculated by POVME 3.0; the center of mass is defined by the ligand (danuglipron) from the initial frame of the structure. The volume is calculated using POVME 3.0. Specific motions are indicated by arrows. In replica 1, the ligand shifts upward, suggesting the potential dissociation from the receptor. In replica 2, the initial tight binding of ECL2 and ECL3 shows a progressive loss of the interaction network, characterized by an opening of the binding pocket.

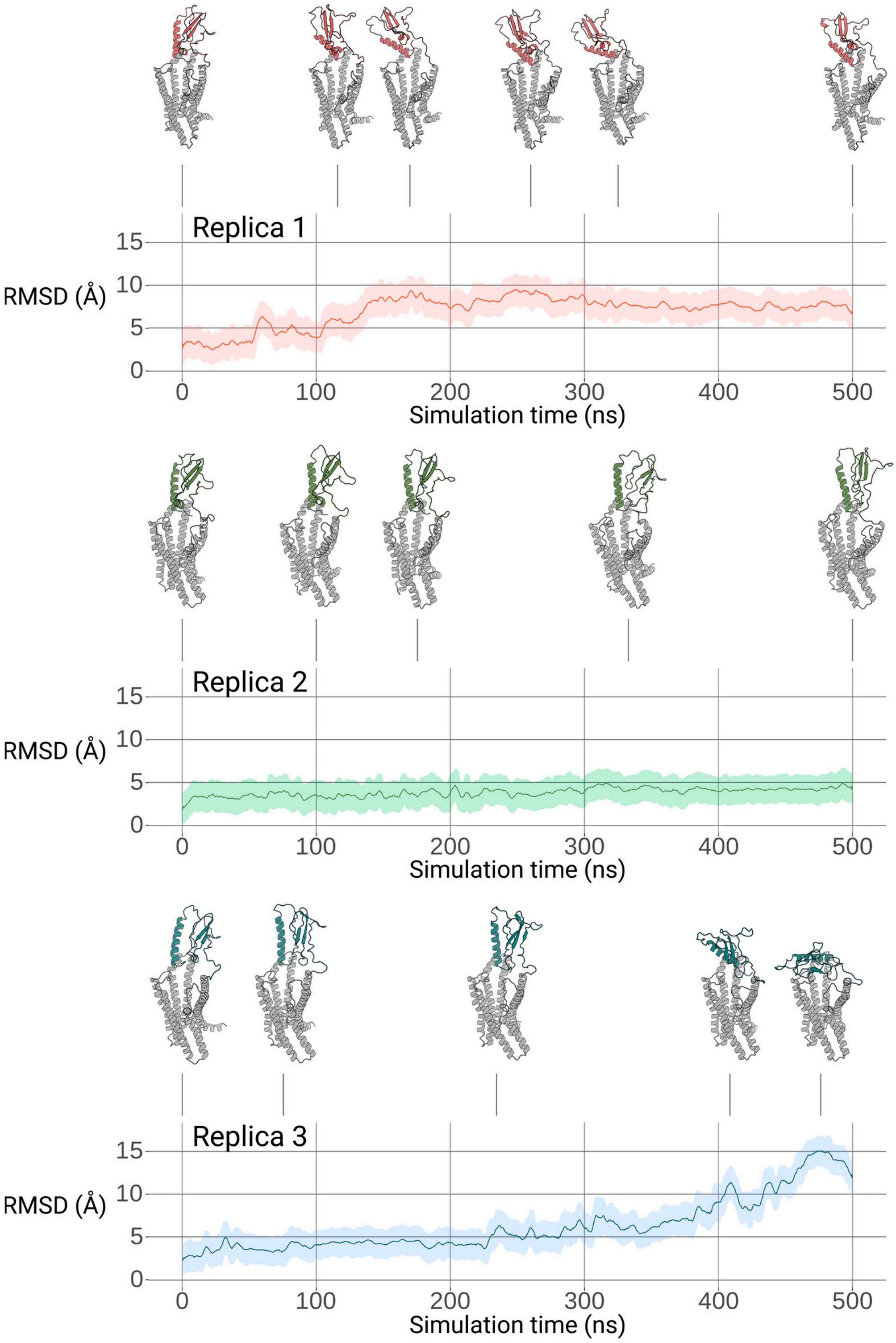

Figure S3. Structural dynamics of ligand-free GLP-1R by MD simulation. The conformation of the ECD adopts distinct shapes across different replica simulations. In replica 1, the ECD is oriented towards ECL2, whereas in replica 3, the ECD tilts forward within the binding pocket.

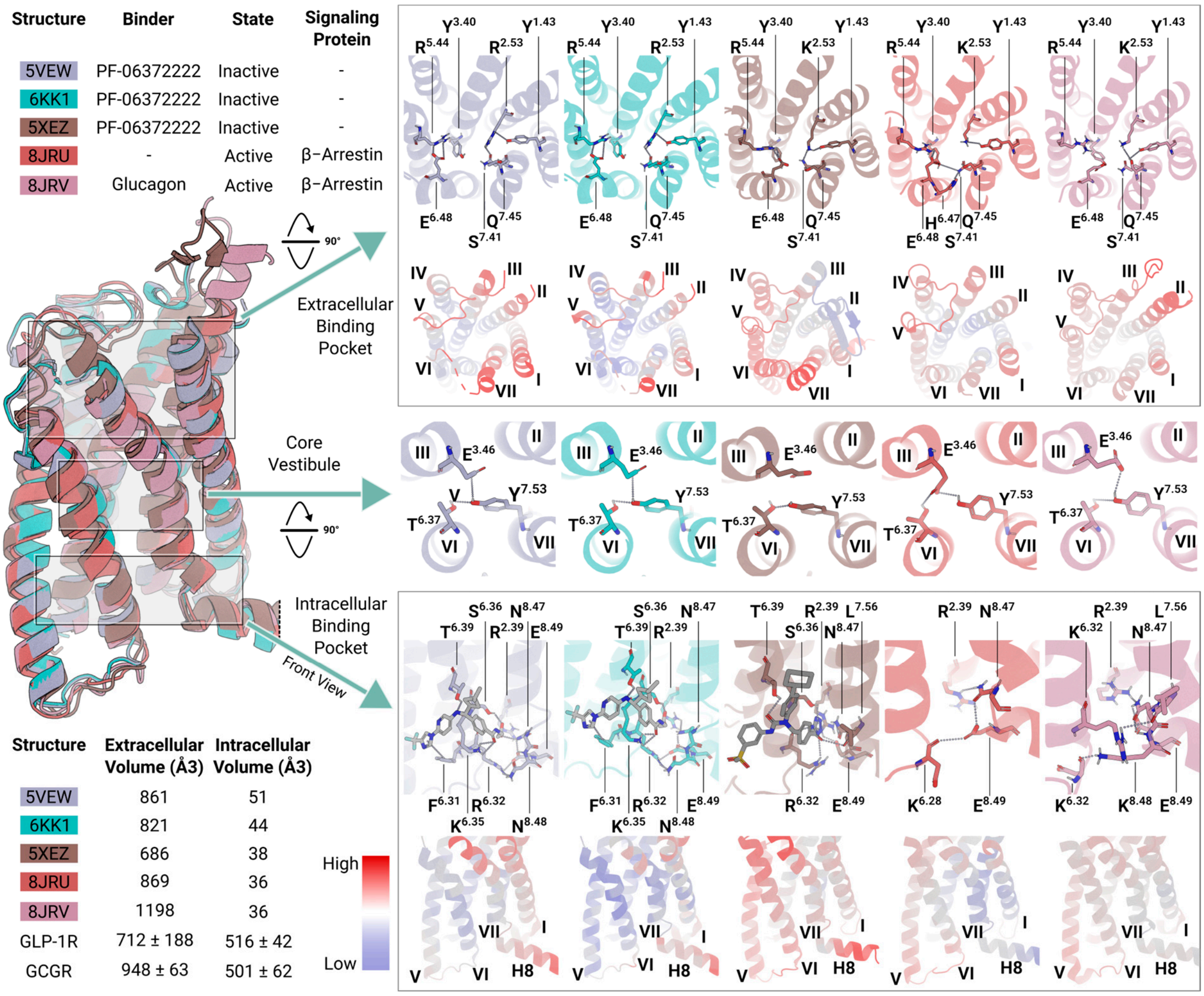

Figure S4. Comparison of β-arrestin-bound and inactive state incretin structures. Visualization of the internal interaction network and structural disorder in the extracellular and intracellular binding pockets, as well as the core vestibule. Five structures were selected: 5VEW and 6KK1 represent the inactive state of GLP-1R, 5XEZ is the inactive structure of GCGR, and 8JRU and 8JRV correspond to the β-arrestin-bound structure of GCGR.

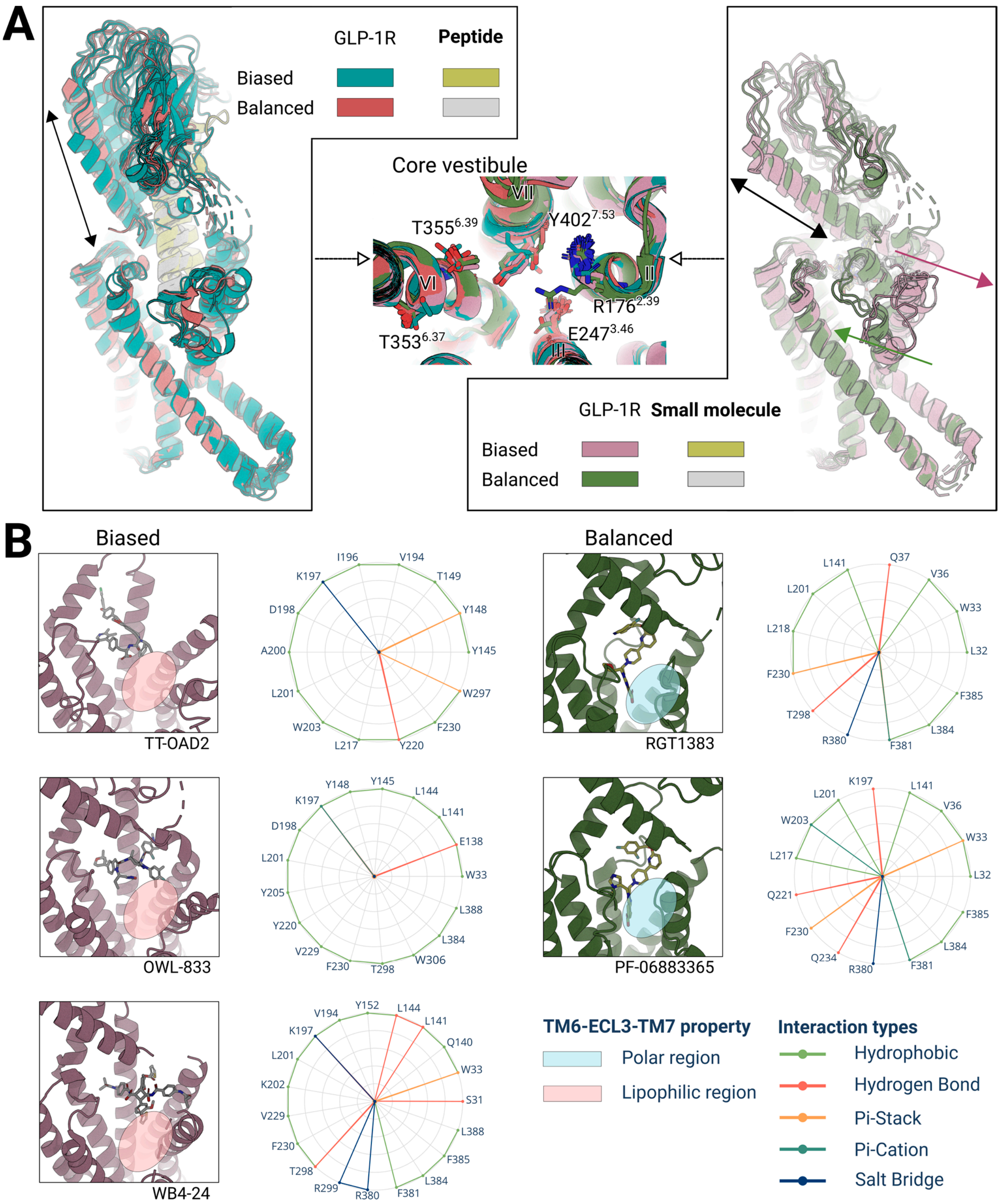

Figure S5. Comparison of biased and balanced agonist-bound GLP-1R structures. A) Alignment of different agonist-bound GLP-1R structures. Balanced small molecules induce a closure of the binding pocket. The black arrow indicates the positional shift in the ECD helix, while green and pink arrows illustrate the conformational movement of the ECL3 relative to the GLP-1-bound reference structure. B) Interaction fingerprint of biased and balanced small molecules in the binding pocket. Both types of ligands induce distinct polarity environment in the TM6-ECL3-TM7 region. Pink circles denote hydrophobic interaction zones, whereas blue circles highlight polar interaction environments.

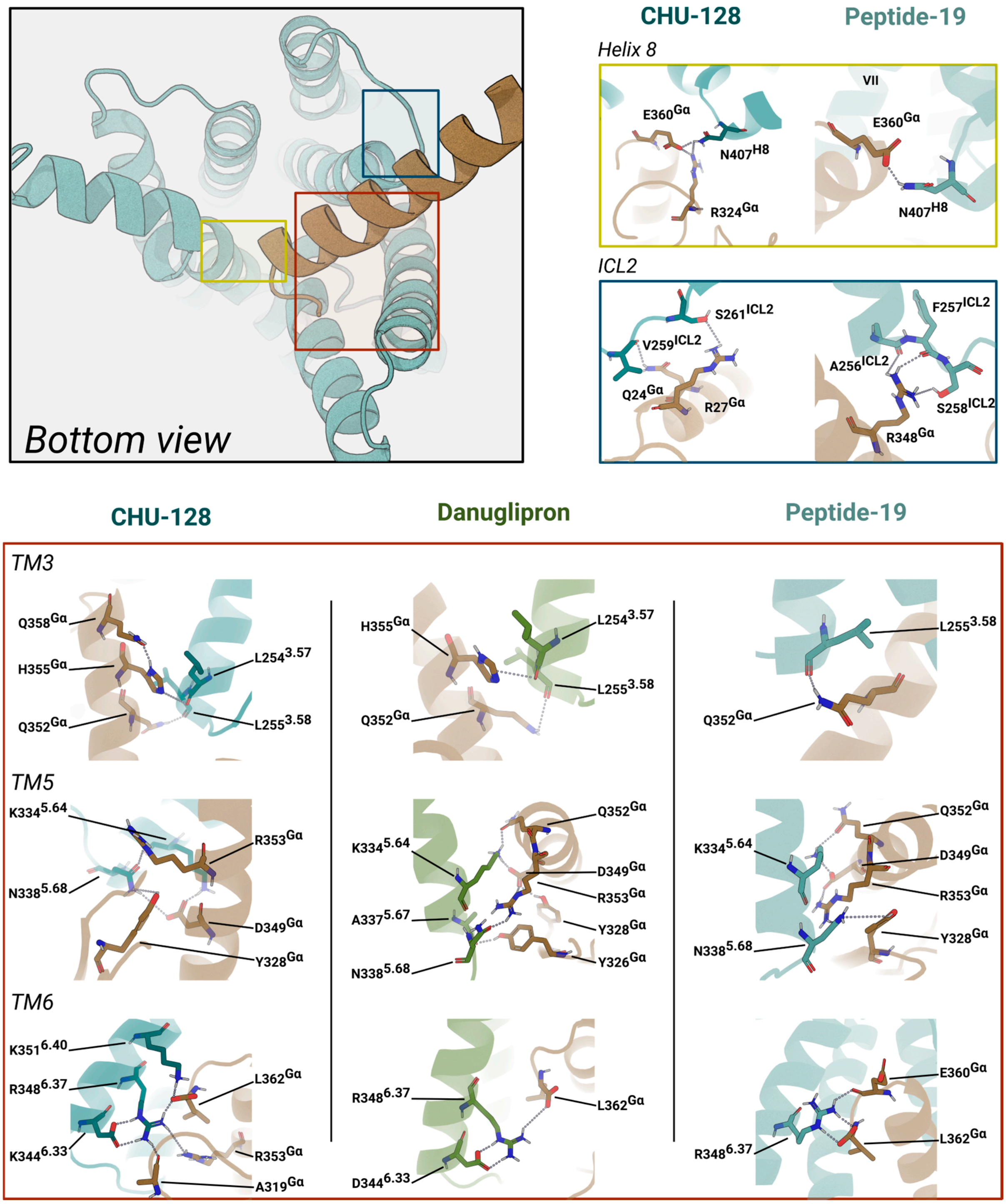

Figure S6. Distinct conformational transitions in the intracellular signaling pocket upon extracellular binding of different ligands to GLP-1R. Visualization of the G protein binding mode in the intracellular G protein binding pocket. During MD simulation of the GLP-1R/danuglipron/G protein complex, the G protein lost its interaction with ICL2 and H8 of the receptor.
